# The impact of SEC23A on 5-FU chemotherapy sensitivity and its involvement in endoplasmic reticulum stress-induced apoptosis in colorectal cancer

**DOI:** 10.1007/s10495-025-02084-2

**Published:** 2025-02-04

**Authors:** Zhaoran Su, Menglan Liu, Mathias Krohn, Sandra Schwarz, Michael Linnebacher

**Affiliations:** 1 Department of Gastrointestinal Surgery, People’s Hospital of Tongling City, Tongling, 244000 China; 2 Molecular Oncology and Immunotherapy, Clinic of General Surgery, University Medical Center Rostock, 18057 Rostock, Germany

**Keywords:** SEC23A, CRC, 5-Fluorouracil, Apoptosis, Drug Resistance, Endoplasmic reticulum stress, Misfolded protein

## Abstract

Background: Colorectal cancer (CRC) represents a significant global health burden, with chemotherapy resistance representing a significant challenge to effective treatment. SEC23A, a core component of the COPII vesicle trafficking system, is of critical importance with regard to protein transport and cellular homeostasis. Nevertheless, its function in CRC progression and chemoresistance remains uncertain. The present study investigates the correlation between SEC23A expression and sensitivity to 5-fluorouracil (5-FU), a widely used chemotherapeutic agent, with particular emphasis on ER stress-induced apoptosis. Methods: A bioinformatic analysis was conducted to evaluate SEC23A expression in CRC and its association with patient prognosis. Chemotherapy sensitivity was predicted using GDSC data and validated experimentally using CRC cell lines with manipulated SEC23A expression. In order to explore the role of SEC23A in acquired drug resistance, patient-derived xenograft (PDX) models and 5-FU-resistant cell lines were employed. Apoptosis assays, cell cycle analysis, and ER stress modulation experiments were performed to elucidate the underlying mechanisms. Results: SEC23A expression was significantly reduced in CRC samples compared to normal tissues. This reduction was linked to a poorer prognosis, including both overall and disease-specific survival. A correlation was observed between low SEC23A expression and increased resistance to 5-FU, as evidenced by both bioinformatic predictions and in vitro experiments. In PDX models, metastatic lesions exhibited decreased SEC23A expression following 5-FU treatment in comparison to primary tumors. Overexpression of SEC23A in 5-FU-resistant cell lines restored sensitivity to the drug and increased apoptosis. Bioinformatic and experimental analyses revealed a robust correlation between SEC23A and ER stress-related apoptotic pathways. Elevated expression of SEC23A was observed to facilitate the accumulation of misfolded proteins in response to 5-FU treatment, which in turn resulted in increased ER stress and apoptosis. Conclusions: SEC23A plays a crucial role in modulating the sensitivity of CRC cells to 5-FU by regulating ER stress-induced apoptosis. Its downregulation contributes to chemoresistance, indicating that SEC23A may serve as a prognostic marker and therapeutic target in CRC. Strategies aimed at upregulating SEC23A or enhancing ER stress may provide new avenues for overcoming chemoresistance and improving treatment outcomes for CRC patients.

**Supplementary Information:**

The online version contains supplementary material available at 10.1007/s10495-025-02084-2.

## Background

Colorectal cancer (CRC) represnts one of the most significant global health challenges [1]. This malignancy is characterized by an alarming and steadily rising incidence, with both lifestyle factors and aging populations contributing to its prevalence. This underscores the necessity for comprehensive research aimed at elucidating underlying molecular mechanisms and developing innovative therapeutic strategies that can effectively combat CRC [2].

Among the various treatment modalities, chemotherapy plays a pivotal role, with 5-fluorouracil (5-FU) being one of the most widely used agents [3–5]. This drug has been a cornerstone of CRC treatment due to its ability to inhibit thymidylate synthase, a key enzyme required for DNA synthesis and repair, thereby impeding tumor growth [6–7]. However, despite the initial success of chemotherapy, the emergence of resistance to chemotherapeutic agents like 5-FU represents a significant challenge, undermining long-term clinical outcomes and leading to treatment failure in many CRC patients [8–9]. Consequently, the investigation of the mechanisms underlying chemotherapy resistance has become a primary focus of contemporary research. A deeper understanding of these mechanisms is crucial for enhancing the efficacy of current treatments and developing novel therapeutic strategies that can circumvent resistance and improve patient survival.

Section 23 homolog A (SEC23A) is a fundamental component of the cellular machinery responsible for transporting proteins from the endoplasmic reticulum (ER) to the Golgi apparatus, a process vital for maintaining cellular function and homeostasis [10]. Specifically, SEC23A functions as a subunit of the coat protein complex II (COPII), which is essential for the formation of transport vesicles that ferry newly synthesized proteins from the ER to their destinations within the cell.

In recent years, the function of SEC23A in central processes of tumor development and metastasis, including tumor cell proliferation, migration, and invasion has begun to be elucidated [11–13]. Moreover, SEC23A has been identified as a principal regulator of the ER stress response, a cellular process that plays a pivotal role in the survival and drug resistance of tumor cells [14]. The ER stress response is initiated in response to cellular stressors, such as chemotherapy, and can result in diverse outcomes, including apoptosis or adaptation to the stressor. The dual function of SEC23A in both vesicle transport and the stress response suggests that it may be a pivotal factor in determining the fate of cancer cells under chemotherapeutic stress.

The primary objective of this study was to examine the complex relationship between SEC23A expression and the sensitivity and resistance of CRC cells to 5-FU chemotherapy. By investigating the molecular mechanisms through which SEC23A affects chemotherapy response, we sought to elucidate its potential role in mediating ER stress-induced apoptosis in CRC. This could provide valuable insights into the manner by which SEC23A influences the outcome of chemotherapeutic treatment by modulating the balance between cell survival and death. Moreover, a more profound comprehension of SEC23A’s function in CRC could facilitate the creation of targeted therapies that capitalize on its role in apoptosis, thereby enabling a more personalized approach to treatment.

## Methods

### Bioinformatics analysis

#### Analysis of *SEC23A* gene expression in pan-cancer and COAD

Two comprehensive visualization bioinformatics tools based on the R language were utilized for the analysis: Xiantao Academic [15] and HOME for Researchers [16]. Whole transcriptome sequencing data of 33 cancer entities and their corresponding normal organ tissues were obtained from the Cancer Genome Atlas (TCGA) [17] and the Genotype-Tissue Expression (GTEx) databases [18]. Subsequently, the mRNA expression levels of *SEC23A* were analyzed in both pan-cancer and colon adenocarcinoma (COAD) using paired and unpaired analyses.

#### Immunohistochemical analysis

The Human Protein Atlas (HPA) [19] was employed to examine the differential expression of SEC23A in CRC tissues and normal colonic mucosa tissues, based on immunohistochemical data.

#### Single-cell analysis

Raw single-cell transcriptome sequencing data from a pair of CRC and matched normal tissues (GSM4904240, GSM4904239) were obtained from the Gene Expression Omnibus data base (GSE161277). The Seurat (v3.0.2) package was employed to create the object, to filter out and exclude low-quality cells and to perform standard data preprocessing. The remaining cells were subsequently subjected to analysis. To normalize the library size effect across cells, UMI counts were scaled using a scale factor of 10,000. Subsequently, data were log-transformed, and the ScaleData function in Seurat was employed to perform variation regression on factors including “percent.mt,” “nCount_RNA,” and “nFeature_RNA.” Variable genes were subjected to principal component analysis for uniform manifold approximation and projection (UMAP) visualization and clustering. Furthermore, the expression levels of SEC23A were examined in diverse cellular populations.

#### Prognosis analysis

The datasets of 643 CRC patients, comprising clinical details and gene expression values, were obtained from TCGA. The univariate analysis employed the log-rank method to examine the relationship between *SEC23A* expression (median-based) and overall survival (OS), disease-specific survival (DSS), and progression-free interval (PFI). To identify risk factors for OS, a Cox regression analysis was conducted on 290 patients with complete clinicopathological data, including OS, age, gender, weight, pathologic T, N, M stages, and *SEC23A* expression levels. Variables with a univariate analysis *P*-value < 0.100 were included in the multivariate analysis. Subsequently, those variables with a *P*-value < 0.050 were identified as the final risk factors for OS. A nomogram was utilized to facilitate the visual representation of the results.

#### Co-expression and enrichment analysis

The Gene Expression Profiling Interactive Analysis 2 (GEPIA2) online database tool [20] was utilized to identify the top 100 genes co-expressed with SEC23A in COAD. An enrichment analysis, including Gene Ontology (GO) and Kyoto Encyclopedia of Genes and Genomes (KEGG) pathway analysis, was conducted using the clusterProfiler package in R (version 4.0.3). A gene set enrichment analysis (GSEA) was performed on TCGA-COAD data using the GSEA software (version 4.3.0). The analysis employed the c2.cp.v7.2.symbols.gmt gene set file. To ensure the reliability of the results, a total of 5,000 permutations were performed. Significant enrichment was determined according to the following criteria: a normalized enrichment score (NES) > 2, false discovery rate (FDR) q-value < 0.05, and an adjusted *P*-value (*P*. adjust) < 0.05.

#### Prediction of chemotherapy drug sensitivity

The RNA-sequencing expression profiles (level 3) and corresponding clinical information were obtained from the TCGA COAD dataset (accessed on June 15, 2023). The chemotherapeutic response was predicted for each sample based on the Genomics of Drug Sensitivity in Cancer (GDSC) database [21] using the R package “pRRophetic.” The half-maximal inhibitory concentration (IC_50_) of the samples was estimated by ridge regression. All parameters were set to their default values. The batch effect of Combat and the tissue type of all tissues were used to average the duplicate gene expression.

### Cell culture and induction of drug-resistant cell lines

Two CRC cell lines were selected for further analysis; HROC285 T0 M2 and HROC110 T1 M7. All lines have been subjected to authentication by short tandem repeat analysis and were mycoplasma negative [22]. Cells were cultivated in DMEM-F12 (Catalog No. DMEM-12-A, Capricorn-Scientific, Ebsdorfergrund, Germany), 2 mM L-glutamine, 10% fetal calf serum and maintained in an incubator containing 5% CO_2_ at 37 °C. 5-FU resistance was achieved by continuously exposing the cells to stepwise increasing concentrations of 5-FU (steps: 0.1 µM, 0.5 µM, 1 µM, and 5 µM), with each exposure lasting approximately 1–3 days. When a significant number of cells died or cell division was notably slow, the increase in 5-FU concentration was postponed. Once the resistance index (RI) was ≥ 10 (with RI = IC_50_ (resistant cell line) / IC_50_ (original cell line), they were classified as 5-FU-resistant and designated as HROC285 T0 M2/5-FURE and HROC110 T1 M7/5-FURE, and used for subsequent experiments.

### Lentiviral infection

Commercially lentiviral particles (Supplementary Material 1) for SEC23A overexpression, SEC23A knockdown (shRNA), and corresponding control vectors (empty vector and non-targeting shRNA) were used to infect HROC285 T0 M2 and HROC110 T1 M7. Cells were seeded at a density of 1 × 10^5^ cells per well, culture medium was replaced the following day with 2 ml of fresh medium containing 6 µg/ml polybrene (Catalog No. TR-1003, Merck, Darmstadt, Germany), followed by the addition of an appropriate volume of lentiviral suspension, and incubation at 37 °C. Green fluorescent protein (GFP) was utilized to evaluate transduction efficiency through flow cytometry (FC). Puromycin selection was performed when necessary to ensure transduction efficiency ≥ 80% (Supplementary Material 2).

### mRNA extraction, reverse transcription and qRT-PCR assay

Total RNA was extracted (Total RNA Kit, Catalog No. 0621018-25, VWR, Germany) and 1 µg was reverse transcribed into cDNA. The qRT-PCR assay was conducted using TaqMan probes (Supplementary Material 1), and the relative expression levels were analyzed based on the threshold cycle (Ct) values, normalized to the endogenous reference gene *GAPDH*, and calculated using the 2^−ΔΔCt^ method.

### Clinical case and patient-derived xenograft (PDX)

A retrospective review of the CRC patients database at our clinic was conducted from 2010 to 2022. The following criteria were employed to identify cases for inclusion in the study: (1) Surgical resection or biopsy of the tumor, providing samples at least twice, and (2) no neoadjuvant therapy prior to the initial tissue removal. The exclusion criteria were as follows: (1) age below 18 years, (2) concurrent other malignant tumors, and (3) failure in patient-derived xenograft (PDX) establishment, as previously reported [23].

### Protein extraction and western blot assay

A total of 3 × 10^6^ cells or 50 mg PDX tissue samples were lysed in 500µL RIPA lysis buffer (Catalog No. 89900, Thermo Fischer Scientific, Schwerte, Germany) containing protease inhibitor (Catalog No. 11836170001, Roche, Basel, Switzerland). Protein samples were subjected to SDS-PAGE gel electrophoresis and subsequently transferred onto a nitrocellulose membrane (Catalog No. 1704270, Bio-Rad, Feldkirchen, Germany). Membranes were washed with Tris-buffered saline with 0.1% Tween 20 and blocked with 5% milk, subsequently incubated with the primary antibody over night at 4℃, followed by the secondary antibody for 60 min at room temperature. Vinculin and GAPDH (all antibodies listed in Supplementary Material 1) were used as internal control proteins.

### Cell proliferation and drug sensitivity assay

Logarithmic phase cells were seeded in a 96-well plate (2–4 × 10⁴ cells/well in 100µL). The drugs 5-FU, oxaliplatin, and irinotecan were added after 24 h at the maximum concentrations of 200 µg/mL, 50 µg/mL, and 80 µg/mL, respectively. Additionally, twofold stepwise dilutions to attain eight distinct drug concentrations and controls were added; all in triplicates (Supplementary Material 1). Plates were incubated for 72 h and stained with 0.2% crystal violet. After drying, the dye was solubilized with 1% SDS, and the absorbance was measured at a wavelength of 570 nm. The IC_50_ values were calculated using Prism 9.0 software. Cell growth over time was measured in the absence of pharmacological intervention with cells subjected to staining at 12 h intervals.

### Cell cycle and apoptosis assay

For cell cycle and apoptosis assays, logarithmic phase cells were harvested and fixed in 70% ethanol at 4 °C overnight. Following incubation with 100 µg/mL RNase A at 37 °C for 30 min, cells were stained with propidium iodide (PI, 50 µg/mL) for 30 min. Cell cycle distribution was analyzed by measuring the DNA content by FC to distinguish between G0/G1, S, and G2/M phases. A double-staining with Annexin V-APC and PI (all reagents listed in Supplementary Material 1) was performed for quantification apoptotic cells (Annexin V positive) by FC.

### Colony formation assay

Cells were treated with 50 µg/mL 5-FU for 24 h, washed with PBS to remove any residual 5-FU, trypsinized, and counted. They were re-seeded at a density of 1 × 10^4^ cells per well in a new 6-well plate and cultured for approximately two weeks to allow for colony formation. Resulting colonies were stained with crystal violet and counted.

### Detection of misfolded proteins

Cells were seeded at a density of 5 × 10^5^ cells per well in a 6-well plate. Upon reaching approximately 50% confluency, cells were treated with 500 µg/mL of 5-FU for 24 h, and subsequently fixed with 500µL of -20 °C pre-chilled formaldehyde at 4 °C for 20 min. Cells were permeabilized with 100µL of 1% Triton X-100 for 5min at room temperature, 500µL of 1% Congo Red (Supplementary Material 1) solution was added per well, and the plate was shaken at room temperature for 20 min. The staining intensity was assessed under a reverse microscope and scored with the following points: 0 for negative staining, 1 for pale yellow, 2 for light brown, and 3 for dark brown. The positive area was scored based on the percentage of positive cells: 1 point for 0–25%, 2 for 26–50%, 3 for 51–75%, and 4 for 76–100%. The final score was obtained by adding the staining intensity and positive area scores. This procedure was repeated randomly five times.

### Enhancement and reduction of ER stress

Tunicamycin (TM) was used to induce ER stress, while tauroursodeoxycholic acid (TUCDA) to inhibit ER stress. Following treatment with TM (1 µg/mL for 24 h), TUCDA (50 µg/mL for 24 h), and 5-FU alone, or in combination, cells were harvested and apoptosis was assessed as described before.

### Statistical methods

Analysis was conducted using SPSS 19.0 and R 4.0.3 software. The independent sample t test, paired sample t test, and Mann–Whitney U test were employed for group comparisons. Prognostic evaluation utilized log-rank and multivariate Cox regression analyses. Spearman correlation analysis was applied to examine the relationship between SEC23A expression and co-expressed genes. Statistical significance was set at a *P*-value < 0.05.

## Results

### SEC23A expression and association with CRC prognosis

**Fig. 1:**
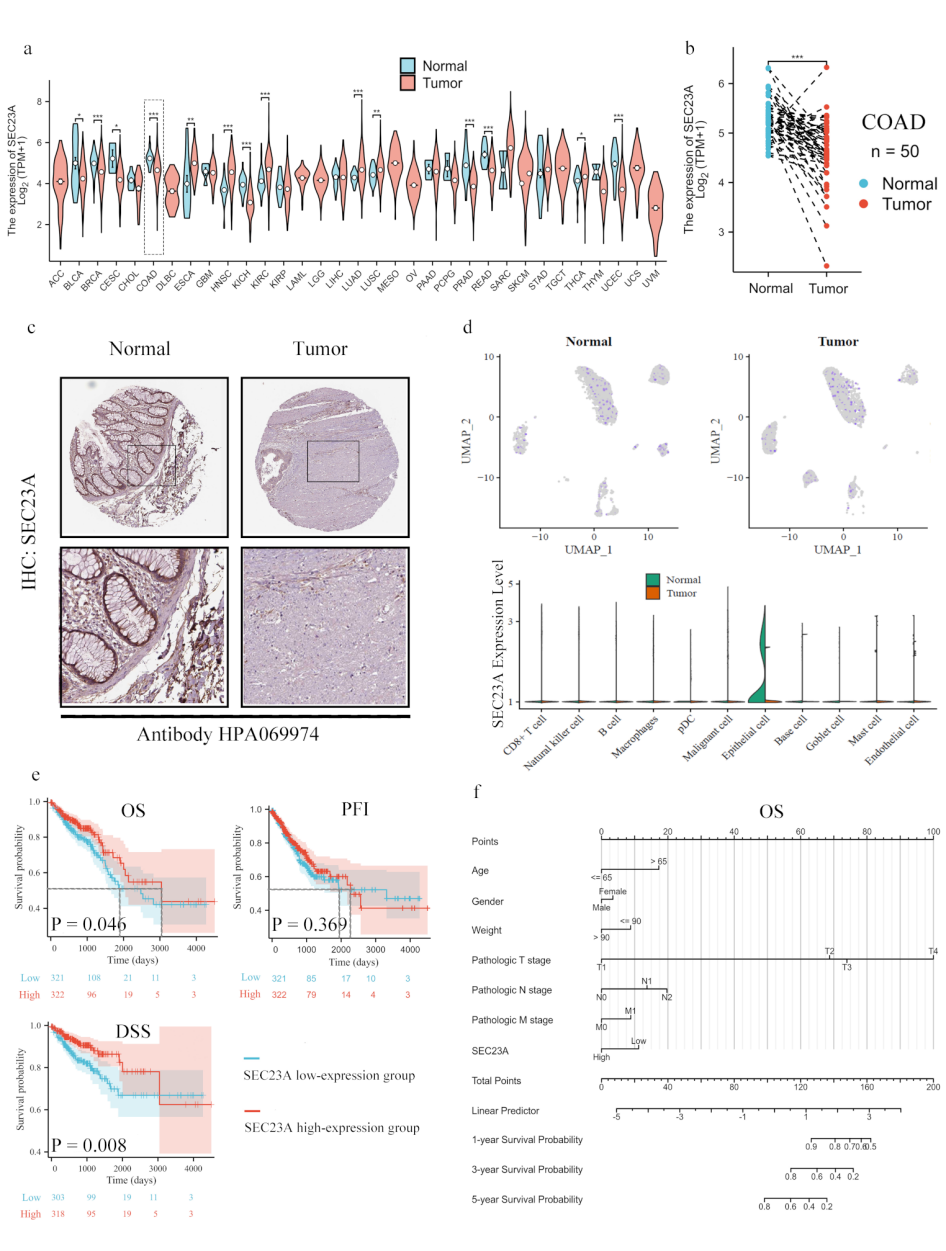
SEC23A expression and association with CRC prognosis. (**a**) *SEC23A* transcript levels across 33 different tumor types from the TCGA and GTEx datasets, highlighting significant down-regulation in COAD compared to normal tissues in unpaired analyses. (**b**) Paired analysis of *SEC23A* expression in COAD showed significantly decreased levels in tumor compared to adjacent normal tissues. (**c**) IHC data from the HPA showed lower SEC23A protein expression in COAD compared to normal tissues. (**d**) Single-cell RNA sequencing data from the GSE161277 dataset showed a significant downregulation of *SEC23A* in epithelial cells within COAD. (**e**) Kaplan-Meier survival analysis illustrates the correlation between low *SEC23A* expression and poor OS and DSS in COAD patients. (**f**) Multivariate cox analysis of *SEC23A* expression and other clinicopathologic factors. Scores: for the individual scores corresponding to each predicted variable. Total score: sum of individual score points. Linear Predictor: weighted sum of variables in the cox regression model with high values indicating worse prognosis. *P value < 0.05, **P value < 0.01, ***P value < 0.001

Significant variations in *SEC23A* transcriptional levels were observed through bioinformatic analysis using data from TCGA and GTEx across 33 different tumor types (Fig. 1a). In particular, in COAD, both unpaired (*P* < 0.001, Fig. 1a) and paired analyses (*P* < 0.001, Fig. 1b) revealed markedly decreased expression of *SEC23A* compared to normal tissues. IHC data from the HPA demonstrated that the SEC23A protein is also expressed at lower levels in cancerous tissues than in adjacent normal tissues (Fig. 1c). Single-cell analysis of the GSE161277 dataset further corroborated the downregulation of *SEC23A* expression in COAD when compared to normal tissues, with the primary occurrence in epithelial cell populations (*P* < 0.001, Fig. 1d and Supplementary Material 3). Kaplan–Meier analysis (Fig. 1e) revealed a significant correlation between decreased SEC23A expression and poor OS (*P* = 0.046) and DSS (*P* = 0.008), but not PSI (*P* = 0.369). Cox regression analysis on 290 COAD patients identified low SEC23A expression (*P* = 0.026), pN stages (N1, *P* = 0.038; N2, *P* = 0.003), and age > 65 years (*P* = 0.002) as independent prognostic factors for poor OS (Fig. 1f; Table 1).

**Table 1:** Cox analysis of the final independent risk factors for overall survival (OS) in 290 COAD patients

Characteristics	Total(*N*)	Univariate analysis		Multivariate analysis	
		Hazard ratio (95% CI)	*P* value	Hazard ratio (95% CI)	*P* value
Pathologic T stage	290		< 0.001		
T1	6	Reference		Reference	
T2	45	5411601.8797 (0.000 - Inf)	0.996	4770713.6162 (0.000 - Inf)	0.997
T3	206	11951581.6951 (0.000 - Inf)	0.996	6878109.5076 (0.000 - Inf)	0.996
T4	33	40863162.1326 (0.000 - Inf)	0.996	25528135.9271 (0.000 - Inf)	0.996
Pathologic N stage	290		0.001		
N0	165	Reference		Reference	
N1	75	1.932 (1.035–3.607)	0.039	2.086 (1.042–4.177)	**0.038**
N2	50	3.132 (1.694–5.792)	< 0.001	2.866 (1.434–5.730)	**0.003**
Pathologic M stage	290		0.003		
M0	247	Reference		Reference	
M1	43	2.592 (1.451–4.631)	0.001	1.500 (0.774–2.906)	0.230
Gender	290		0.884		
Female	138	Reference			
Male	152	0.963 (0.577–1.606)	0.884		
Age (years)	290		0.007		
<= 65	143	Reference		Reference	
> 65	147	2.079 (1.194–3.620)	0.010	2.504 (1.414–4.431)	**0.002**
SEC23A	290		0.069		
Low	120	Reference		Reference	
High	170	0.616 (0.364–1.043)	0.071	0.549 (0.323–0.932)	**0.026**
Weight (Kg)	290		0.111		
<= 90	212	Reference			
> 90	78	0.565 (0.267–1.197)	0.136		

Cox regression analysis on 290 COAD patients identified low SEC23A expression (*P* = 0.026), pN stages (N1, *P* = 0.038; N2, *P* = 0.003), and age > 65 years (*P* = 0.002) as independent prognostic factors for poor OS

### SEC23A expression correlates with chemotherapy sensitivity

**Fig. 2:**
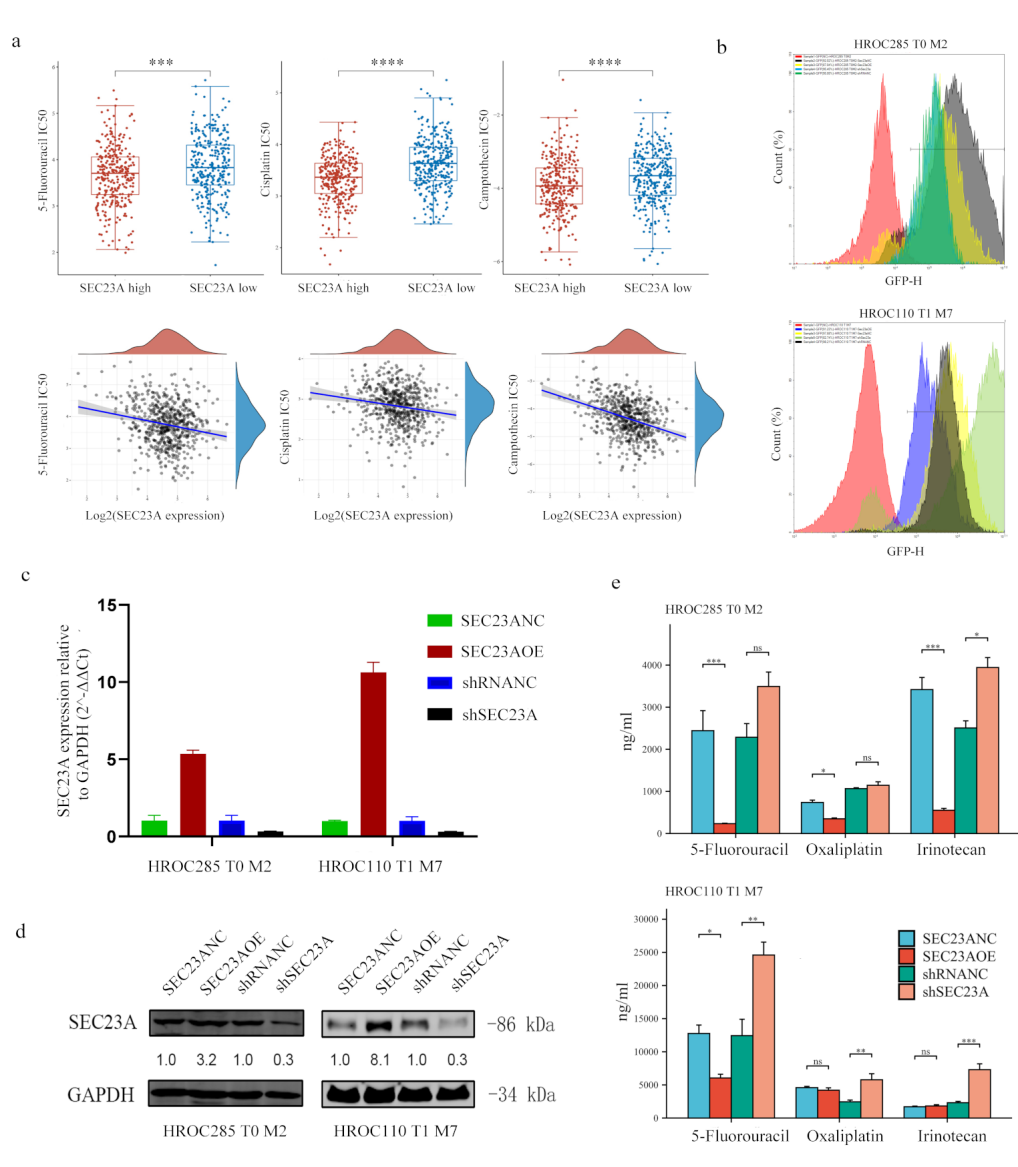
SEC23A expression correlates with chemotherapy sensitivity in CRC. (**a**) Bioinformatic analysis showed a significant correlation between *SEC23A* expression and sensitivity to 5-FU, cisplatin and camptothecin, with lower IC_50_ values in the high expression groups. (**b**) Lentiviral transduction efficiency of the subclones was verified by FC, with all transfection rates exceeding 80%. (**c, d**) Establishment and validation of SEC23A overexpression and knockdown in CRC cell lines HROC285 T0 M2 and HROC110 T1 M7 by lentiviral transduction, confirmed by qRT-PCR and WB. (**e**) IC_50_ values of 5-FU, oxaliplatin and irinotecan of CRC cell lines with different SEC23A expression levels. *P value < 0.05, **P value < 0.01, ***P value < 0.001

Bioinformatic chemotherapy drug sensitivity prediction based on TCGA and GDSC revealed a statistically significant correlation between *SEC23A* expression levels and sensitivity to 5-FU (*P* < 0.001), cisplatin (*P* < 0.001), and camptothecin (the precursor of irinotecan, *P* < 0.001) treatment. The IC_50_ was found to be significantly reduced in the high-expression compared to the low-expression group (Fig. 2a). By lentiviral transduction, cell lines exhibiting overexpression and knockdown of SEC23A were generated for HROC285 T0 M2 (HROC285 T0 M2-SEC23AOE, HROC285 T0 M2-shSEC23A) and HROC110 T1 M7 (HROC110 T1 M7-SEC23AOE, HROC110 T1 M7-shSEC23A) together with the corresponding control cell lines HROC285 T0 M2-SEC23ANC, HROC285 T0 M2-shRNANC, HROC110 T1 M7-SEC23ANC, and HROC110 T1 M7-shRNANC. A transduction efficiency of over 80% was achieved (Fig. 2b) and efficacy of SEC23A overexpression and knockdown was confirmed by qRT-PCR (Fig. 2c) and WB analysis (Fig. 2d). Subsequently, the sensitivity of the two cell lines with varying SEC23A expression levels to three CRC first-line chemotherapeutics was tested (Supplementary Materials 4 and 5). As illustrated in Fig. 2e, the in vitro drug sensitivity results exhibited a high degree of concordance with those of the bioinformatics analysis. In particular, the data indicated that cells with elevated SEC23A expression demonstrated markedly reduced IC_50_ values for the chemotherapeutics. This correlation between drug sensitivity and SEC23A expression was particularly evident in the case of 5-FU (Fig. 2e).

### SEC23A downregulation triggers 5-FU resistance

**Fig. 3:**
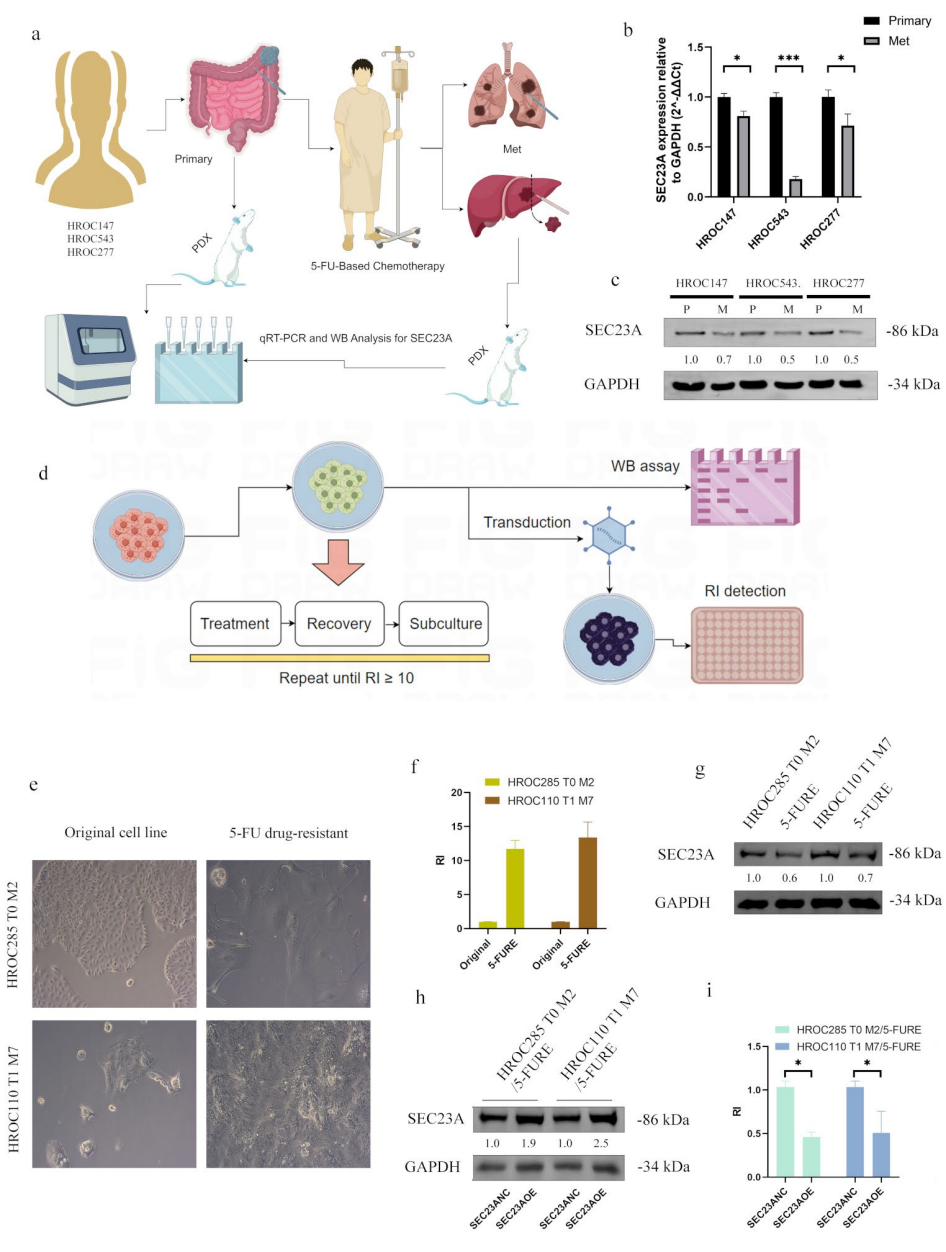
Involvement of SEC23A downregulation in 5-FU resistance. (**a**) Flowchart showing the collection of primary and post-treatment CRC samples and the establishment of PDX models. (**b, c**) SEC23A expression analysis in primary versus metastatic tumor tissues from PDX models showed significantly lower levels in metastatic lesions after 5-FU treatment. (**d**) Flowchart illustrating the establishment of HROC285 T0 M2 and HROC110 T1 M7 5-FU resistant cell lines, assessment of resistance index (RI) and SEC23A expression recovery. (**e**) Development of 5-FU resistant CRC cell lines HROC285 T0 M2/5-FURE and HROC110 T1 M7/5-FURE with associated morphological alterations. (**f**) Drug sensitivity assays confirmed a high resistance index (RI) in the 5-FU-resistant compared to the original cell lines. (**g**) WB analysis showed decreased SEC23A levels in resistant cell lines. (**i, h**) Increased drug sensitivity after re-overexpression of SEC23A in 5-FU-resistant cell lines. *P value < 0.05, **P value < 0.01, ***P value < 0.001

Primary and metastatic tumor samples from three CRC patients were collected and subsequently used to establish corresponding PDX models. Figure 3a illustrates that the tissues from all three primary lesions originated from the colon, while the paired metastatic lesions originated from the liver (in two cases) or the lung (in one case). SEC23A expression was markedly reduced in metastatic lesions following 5-FU treatment in comparison to the primary tumors (Fig. 3b and c). Figure 3d illustrates how two resistant cell lines, HROC285 T0 M2/5-FURE and HROC110 T1 M7/5-FURE, were developed by exposing them to increasing concentrations of 5-FU. These resistant lines displayed notable morphological alterations (Fig. 3e), exhibited an RI value exceeding 10 (Fig. 3f), and expressed markedly diminished SEC23A levels when compared to the original lines (Fig. 3g). The rescue of SEC23A overexpression in the two resistant cell lines (HROC285 T0 M2/5-FURE-SEC23AOE and HROC110 T1 M7/5-FUR-SEC23AOE) by lentiviral transfection was successful, as confirmed by WB analysis (Fig. 3h). The sensitivity to 5-FU increased significantly in both cell lines (*P* < 0.05, Fig. 3i and Supplementary Material 6) when SEC23A was overexpressed.

### High SEC23A promotes 5-FU-induced apoptosis

**Fig. 4:**
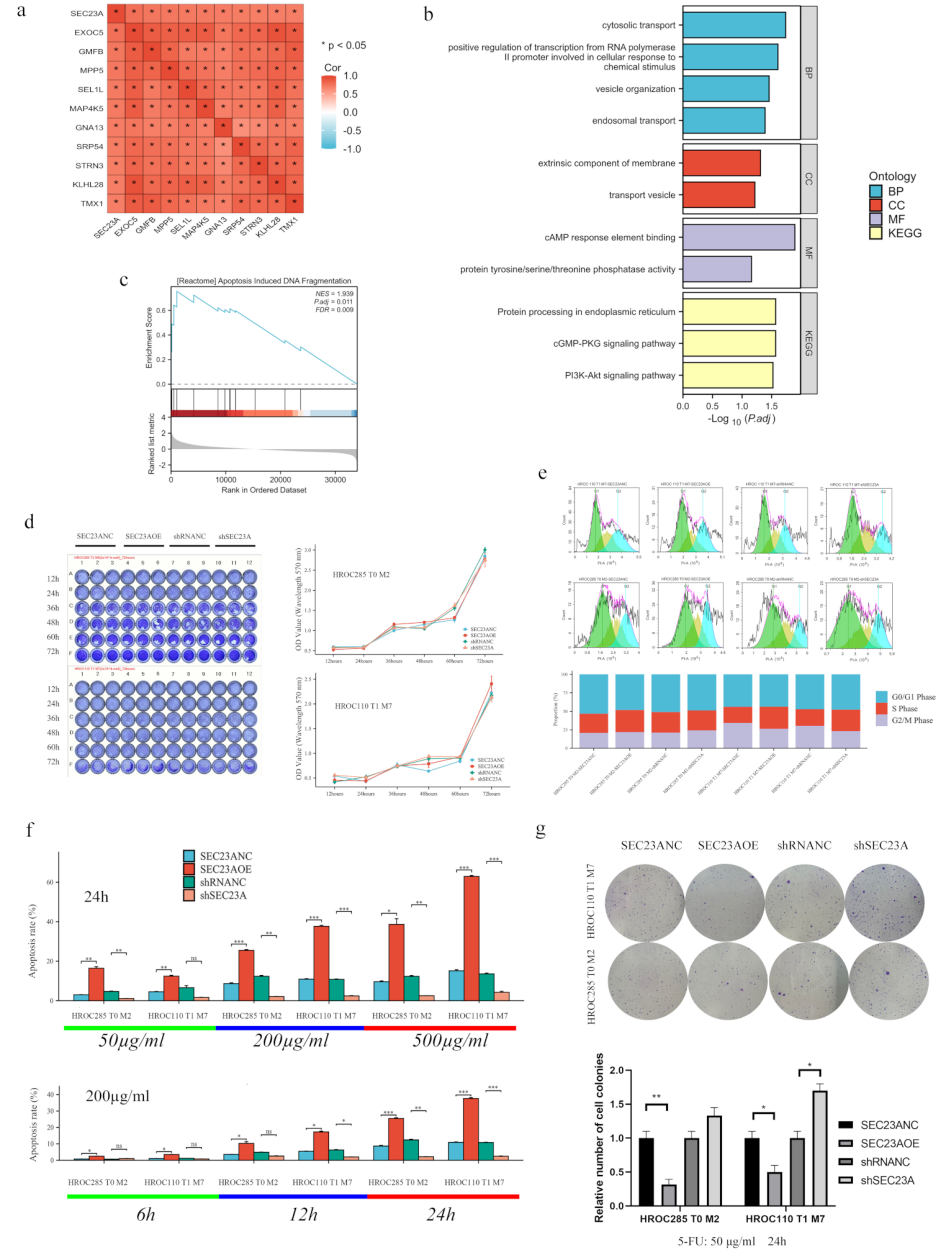
High SEC23A promotes 5-FU-induced apoptosis. (**a**) Identification of the top 10 genes co-expressed with SEC23A in CRC by bioinformatic analysis. (**b**) KEGG and GO pathway analysis revealed enrichment of biological processes, cellular components, and molecular functions associated with SEC23A in CRC. (**c**) GSEA highlighted the association between SEC23A and apoptosis-related pathways in CRC. (**d**) Cell proliferation assays showed no significant differences in growth rates between CRC cells with high and low SEC23A expression. (**e**) Cell proliferation assays showed no significant differences in cell cycle distribution between the SEC23A expression groups. (**f**) Apoptosis assays showing a concentration- and time-dependent increase in apoptosis in cells with high SEC23A expression after 5-FU treatment. (**g**) Clonogenic assay results showing a significant reduction in the number of colonies after 5-FU treatment formed by cells with high compared to cells with low SEC23A expression. *P value < 0.05, **P value < 0.01, ***P value < 0.001

The top 100 genes co-expressed with SEC23A were identified through bioinformatics analysis (Supplementary Material 7). The top 10 co-expressed genes were *EXOC5*, *GMFB*, *MPP5*, *SEL1L*, *MAP4K5*, *GNA13*, *SRP54*, *STRN3*, *KLHL28*, and *TMX1* (Fig. 4a). KEGG-GO analyses revealed the pathways and processes associated with SEC23A. The GO analysis revealed that “cytosolic transport,” “positive regulation of transcription,” “vesicle organization,” and “endosomal transport” were the most prominent terms. The GO cellular component analysis revealed a notable enrichment for the terms “extrinsic component of membrane” and “transport vesicle.” The GO molecular function analysis revealed that the terms “cAMP response element binding” and “protein tyrosine/serine/threonine phosphatase activity” exhibited a high degree of enrichment. KEGG pathway analysis showed “protein processing in endoplasmic reticulum,” “cGMP-PKG signaling pathway,” and “PI3K-Akt signaling pathway” were significantly enriched (Fig. 4b). Next, GSEA was performed to elucidate the functional significance of SEC23A in CRC. SEC23A expression was associated with apoptosis-related pathways (Fig. 4c). We tested the effect of different SEC23A levels on CRC cell growth using cell proliferation assays. No significant differences in cell proliferation were observed between high and low SEC23A expression groups (Fig. 4d). Cell cycle distribution analysis showed no statistically significant differences in cell cycle distribution between SEC23A expression groups (Fig. 4e). Apoptosis assays determined SEC23A’s effect on 5-FU-induced apoptosis at different concentrations and time points. As shown in Fig. 4f, g, and Supplementary Material 8, cells expressing high levels of SEC23A demonstrated an increase in apoptosis and a decrease in colony forming capacity after 5-FU treatment.

### SEC23A is involved in the regulation of ER stress-induced apoptosis

**Fig. 5:**
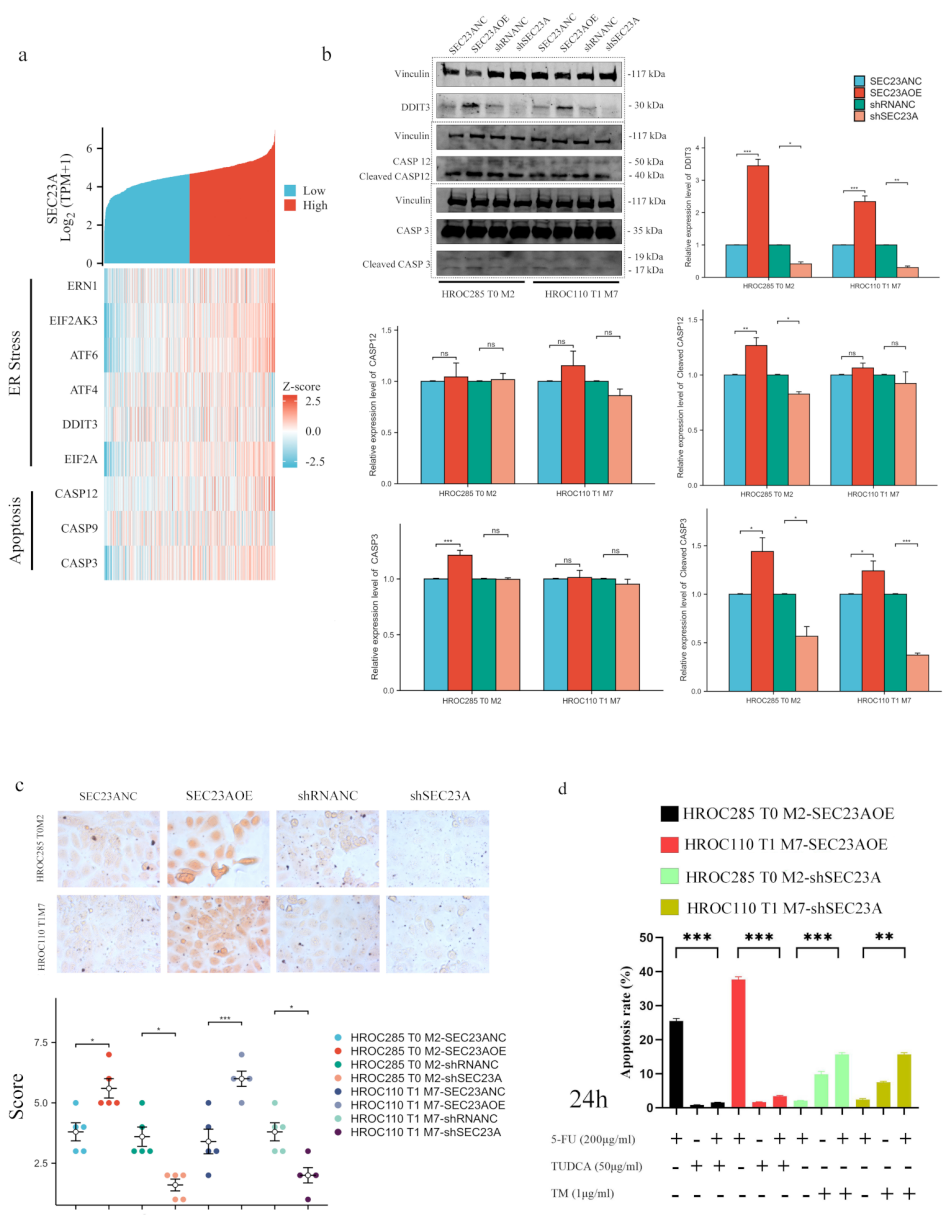
SEC23A involvement in ER stress-induced apoptosis. (**a**) Correlation analysis between *SEC23A* expression and genes involved in ER stress and related apoptotic pathways in CRC. (**b**) WB analysis confirming the association between SEC23A expression and key proteins involved in ER stress-induced apoptosis after 5-FU treatment. (**c**) Congo Red staining showing higher misfolded protein levels after 5-FU treatment in CRC cells with high compared to cells with low SEC23A expression. (**d**) Experimental manipulation of ER stress showing that TM increases while TUDCA decreases the sensitivity of low SEC23A-expressing cells to 5-FU-induced apoptosis. *P value < 0.05, **P value < 0.01, ***P value < 0.001

Bioinformatics analysis revealed a significant positive correlation between SEC23A and all genes involved in ER stress and related apoptotic pathways in CRC (Fig. 5a and Supplementary Material 9). This suggests a critical role for SEC23A in regulation of ER stress-induced apoptosis. WB analysis was performed to validate the expression levels of five key proteins associated with ER stress and apoptosis in CRC cells with varying levels of SEC23A expression after 5-FU treatment (50 µg/mL for 24 h). A positive correlation was observed between SEC23A expression and the levels of these critical proteins (Fig. 5b), further supporting the role of SEC23A in modulating ER stress-induced apoptotic pathways. Cells with high SEC23A expression exhibited significantly higher levels of misfolded proteins after 5-FU treatment compared to control groups in Congo red staining assays. In contrast, cells with low SEC23A expression demonstrated a reduction in misfolded protein levels under the same conditions, indicating that SEC23A expression may influence protein folding and aggregation in response to chemotherapy-induced stress (Fig. 5c). Finally, we used pharmacological agents commonly used for this specific purpose to alter the levels of endoplasmic reticulum (ER) stress. TM, an ER stress-activating drug, increased the sensitivity of low SEC23A-expressing CRC cells to 5-FU-induced apoptosis. In contrast, TUDCA, an ER stress inhibitor, decreased this sensitivity (Fig. 5d and Supplementary Material 10).

## Discussion

The present study was conducted to investigate the role of SEC23A in CRC, with a particular focus on its impact on 5-FU chemotherapy sensitivity and ER stress-induced apoptosis. The present study provides new insights into the molecular mechanisms underlying chemoresistance in CRC and highlights the potential of SEC23A as a resistance biomarker and therapeutic target.

SEC23A is most widely recognized for its role in protein trafficking from the ER to the Golgi apparatus, a process critical for maintaining cellular homeostasis [24]. Initial studies found SEC23A to be aberrantly expressed in various cancer types and demonstrated a strong association with tumor progression, metastasis, and drug resistance [25–30]. Despite the paucity of research on SEC23A in CRC, there is evidence to suggest that it may influence proliferation, migration, and invasion, thereby offering potential as a prognostic biomarker [31]. In our study, bioinformatic analysis revealed that SEC23A expression was significantly reduced in CRC compared with normal tissues, and this lower expression significantly correlated with poor OS and DSS. Low SEC23A expression was even an independent risk factor for OS in the COX analysis.

Since the relationship between SEC23A expression and drug sensitivity has been confirmed in several tumor entities [25, 32], we investigated whether this factor also influences CRC patients’ prognosis. The bioinformatic drug sensitivity prediction analysis demonstrated a robust correlation between SEC23A expression and chemo-sensitivity. This could further be experimentally validated with the observation that high SEC23A expression in CRC cells increased their chemo-sensitivity.

Furthermore, significantly diminished SEC23A expression levels were discerned in established CRC PDX models of 5-FU-pretreated metastatic CRC lesions when directly compared to primaries from the same patients, as well as in 5-FU-resistant cell lines. These findings indicate a robust correlation between SEC23A downregulation and the emergence of acquired drug resistance. Reintroduction of SEC23A into resistant cell lines and additional treatment with ER stress inducers restored their 5-FU sensitivity. These findings suggest SEC23A as a potential biomarker predicting 5-FU sensitivity in CRC and therapeutic target for overcoming resistance. The relationship between drug treatment, resistance development, and alterations in SEC23A expression levels in cancer cells has been described previously. Wang et al. [25] proposed docetaxel-induced upregulation of miR-375 followed by decreased SEC23A expression as a resistance development mechanism in prostate cancer. Amodio et al. [33] found that ER stress inducers decreased membrane-bound SEC23A levels in hepatocellular carcinoma cells.

In order to ascertain a functional explanation for the role of SEC23A in mediating chemotherapy sensitivity, we analyzed its influence on cell cycle and cell proliferation. Despite previous research indicating a potential role for SEC23A in specific tumor cell types [13, 29, 34], our findings did not reveal any statistically significant variations in both cell cycle phase distribution and proliferation rates between CRC cells with varying SEC23A expression levels. Other studies demonstrated a role of SEC23A in apoptosis [14, 35]. Our findings revealed a notable correlation between CRC cell apoptosis and SEC23A expression levels following exposure to 5-FU. This observation suggests that SEC23A plays a pivotal role in 5-FU-induced apoptosis.

Considering that many genes co-expressed with SEC23A are associated with ER stress and apoptosis [36–41], and given that co-expressed genes often have analogous functions, we propose that the role of SEC23A in ER protein transport is directly related to the regulation of cellular responses to chemical stimuli. The results of the GO biological process analysis indicate that SEC23A is linked to apoptosis-related pathways in CRC, as evidenced by the enrichment of “cytosolic transport,” “positive regulation of transcription,” and “vesicle transport”. This had functional consequences, as in colony formation assays, 5-FU treatment significantly reduced the number of surviving colonies by high SEC23A-expressing CRC cells, leading us to conclude that SEC23A promotes apoptosis under conditions of high ER stress. The latter is a classical apoptosis inducer, particularly of chemotherapeutics, as misfolded proteins accumulate, thereby triggering the unfolded protein response. Many studies described as malignant tumors’ key mechanism of 5-FU resistance the alleviation of ER stress in order to inhibit apoptosis triggered by ER stress [42–44].

In addition to the established notion that 5-FU kills tumor cells by inhibiting thymidylate synthase and interfering with RNA synthesis, research has also revealed that it can induce ER stress, which further promotes tumor cell apoptosis. Furthermore, the ER stress mechanism plays a significant role in the development of 5-FU resistance [42, 45, 46]. As a crucial component of the COPII vesicle trafficking system, SEC23A plays a vital role in maintaining ER homeostasis by facilitating the transport of correctly folded proteins to the Golgi. Dysregulation of this process could impair ER function and reduce the apoptotic response to chemotherapeutic agents. Amodio et al. [33, 47–48] proposed that in response to ER stress, the reduction of COPII components by the downregulation of SEC23A expression plays a pivotal role in tumor cells’ ER stress response, preventing the accumulation of misfolded proteins in post-ER compartments. This allows these proteins to either refold more efficiently in the ER or be targeted for degradation via the ER-associated degradation pathway and may thus represent an adaptive response of tumor cells when confronted with 5-FU treatment. This mechanism may be relevant to CRC, as we found a significant positive correlation between SEC23A expression and key genes of the ER stress-apoptosis pathway in bioinformatic analysis. Furthermore, the direct link between low SEC23A expression and increased ER stress-related apoptotic response after 5-FU treatment could be experimentally validated.

When proteins are improperly folded in the ER, the accumulation of misfolded proteins leads to the induction of ER stress. If this condition persists and cannot be alleviated by the unfolded protein response, the cell initiates apoptosis [49–51]. Congo red staining results indicated that SEC23A levels affect the accumulation of misfolded proteins, with high SEC23A expressing cells showing more and low SEC23A expressing cells less misfolded proteins after 5-FU treatment. Experimental activation and inhibition of ER stress by specific drugs could further confirm the direct relationship between SEC23A expression, 5-FU-induced apoptosis and ER stress in CRC cells.

**Fig. 6:**
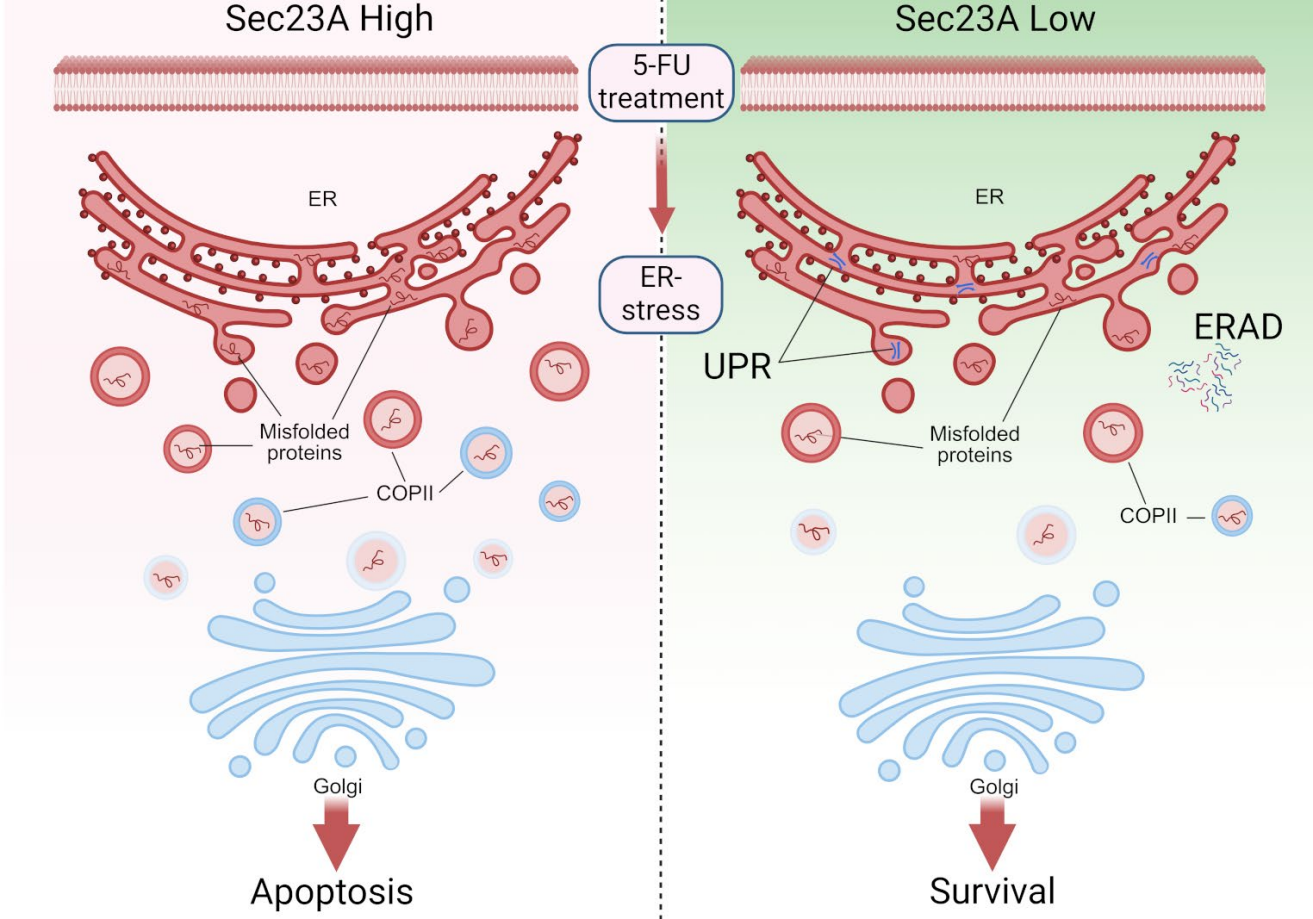
Proposed mechanism schematic. Following exposure to 5-FU, CRC cells downregulate SEC23A, resulting in a reduction of COPII components. These components are of critical importance in managing ER stress by preventing misfolded protein accumulation in post-ER compartments. This regulatory mechanism facilitates the refolding of misfolded proteins within the ER due to the action of the unfolded protein response (UPR) or targeted for degradation via the ER-associated degradation pathway (ERAD). As a result, cells develop increased resistance to ER stress-induced apoptosis, which is a pivotal mechanism underlying drug resistance to 5-FU. Created in https://BioRender.com

To summarize and explain our findings, we propose the following mechanism (Fig. 6): Upon exposure to 5-FU, CRC cells downregulate SEC23A, resulting in a reduction of COPII components. These components are critical in regulating ER stress and preventing the accumulation of misfolded proteins in post-ER compartments. This regulatory mechanism facilitates the efficient refolding of misfolded proteins or their clearance via the ER-associated degradation pathway. This process is more pronounced in cells with low SEC23A expression, whereas such a “self-protective” response is more difficult to initiate in cells with high SEC23A expression.

A limitation of this study is that, although we demonstrated the critical role of SEC23A in modulating CRC sensitivity to 5-FU in vitro, SEC23A, as a key component of the ER budding complex, is involved in numerous essential cellular processes. Its dysregulation could potentially disrupt normal cellular functions. To mitigate potential off-target effects, future investigations should prioritize the development of highly specific targeting strategies, such as tumor-selective delivery systems or approaches that selectively modulate SEC23A expression within tumor cells. Furthermore, preclinical studies utilizing animal models are essential to comprehensively evaluate the therapeutic efficacy and safety of SEC23A-targeted interventions.

## Conclusions

Our findings highlight the significance of SEC23A in CRC, particularly in regard to its function in determining CRC cell sensitivity to 5-FU chemotherapy. SEC23A appears to modulate ER stress-induced apoptosis, and its downregulation contributes to chemotherapy resistance. These findings suggest that SEC23A may serve as a prognostic marker and therapeutic target in CRC. Strategies aimed at upregulating SEC23A or enhancing ER stress may provide new avenues for overcoming chemoresistance and improving patient outcomes in CRC.

## Electronic supplementary material

Below is the link to the electronic supplementary material.

Supplementary Material 1–11

## Data Availability

Some of the data analyzed in this study were obtained from publicly available datasets. They can be found as described in the Materials and Methods. Some of the data presented in this study are available in the article and in the supplementary material.

## Abbreviations

CRC: Colorectal Cancer
5-FU: 5-Fluorouracil
ER: Endoplasmic Reticulum
OS: Overall Survival
DSS: Disease-Specific Survival
PSI: Progression-Free Survival Interval
TCGA: The Cancer Genome Atlas
GTEx: Genotype-Tissue Expression Project
HPA: Human Protein Atlas
PDX: Patient-Derived Xenograft
IC_50_: Half-Maximal Inhibitory Concentration
RI: Resistance Index
GSEA: Gene Set Enrichment Analysis
GO: Gene Ontology
KEGG: Kyoto Encyclopedia of Genes and Genomes
COPII: Coat Protein Complex II
TUDCA: Tauroursodeoxycholic Acid
TM: Tunicamycin
